# Asymptomatic Malaria and Other Infections in Children Adopted from Ethiopia, United States, 2006–2011

**DOI:** 10.3201/eid2107.141933

**Published:** 2015-07

**Authors:** Senait M. Adebo, Judith K. Eckerle, Mary E. Andrews, Cynthia R. Howard, Chandy C. John

**Affiliations:** Children’s Hospital of Philadelphia, Philadelphia, Pennsylvania, USA (S.M. Adebo);; University of Minnesota, Minneapolis, Minnesota, USA (J.K. Eckerle, M.E. Andrews, C.R. Howard, C.C. John);; Indiana University, Indianapolis, Indiana, USA (C.C. John)

**Keywords:** international adoption, infection, Ethiopia, malaria, asymptomatic parasitemia, parasites, vectorborne infections

## Abstract

We screened 52 children adopted from Ethiopia for malaria because they had previously lived in a disease-endemic region or had past or current hepatomegaly or splenomegaly. Seven (13.5%) children had asymptomatic malaria parasitemia by microscopy (n = 2) or PCR (n = 5). Our findings suggest that adoptees at risk for asymptomatic malaria should be screened, preferably by PCR.

International adoptees are at increased risk for infectious diseases (*1*). During 2007–2012, Ethiopia was 1 of the top 5 countries of origin for children who were adopted by persons in the United States (*2*), but few studies have been published on children from Ethiopia who were adopted by persons in the United States (*3*). Malaria caused by *Plasmodium falciparum*, *P. vivax*, and, less frequently, *P. ovale* is endemic to several regions in Ethiopia (*4*). Children adopted from Ethiopia are often living in orphanages in Addis Ababa, an area free of malaria, at the time of their adoption, but they may have lived in a malaria-endemic area before their transfer to the orphanage. The prevalence of asymptomatic malaria parasitemia among these children is not known.

## The Study

We reviewed medical records of all children adopted from Ethiopia and seen at the University of Minnesota International Adoption Clinic (Minneapolis, MN, USA) during February 2006–June 2011 for results of standard infectious disease screening tests recommended by the American Academy of Pediatrics: tuberculosis (by tuberculin skin test or, in children >5 years old, by interferon-γ release assay); intestinal parasites (fecal testing for ova, parasites, and *Giardia intestinalis* antigen); hepatitis B or C virus; HIV; and syphilis (*5*). Children were screened for hepatitis A virus at the discretion of the physician seeing the patient and for malaria by blood smear or PCR if they met screening criteria (i.e., history of living in a malaria-endemic region or a history of or current evidence of splenomegaly or hepatomegaly). The study was reviewed and approved by the University of Minnesota Institutional Review Board.

During the period studied, 255 international adoptees from Ethiopia were seen at the clinic. Adoptees’ mean age at medical evaluation was 2.8 years (range 3.4 months–14.9 years); 148 (58%) were female and 107 (41.9%) were male. All 255 children were asymptomatic for malaria, but 52 met malaria screening criteria and were tested by peripheral blood smear (n = 24), PCR (n = 24), or both (n = 4). Of the 52 children, 7 (13.5%) had blood smear (2 children) or PCR (5 children) results positive for *Plasmodium* species. Table 2 outlines the sensitivity, specificity, and negative and positive predictive values of medical history questions and physical exam signs for asymptomatic malaria. The 2 children with a positive blood smears had low parasite densities (<0.1%), and the species could not be identified. These 2 children were treated before PCR testing was available. Subsequently, PCR became the preferred first-line diagnostic test, and 5 infections were diagnosed on the basis of PCR results: 3 *P. vivax*, 1 *P. falciparum*, and 1 mixed *P. vivax* and *P. falciparum*. Among the 7 children with parasitemia, 2 had a palpable spleen tip, 2 had a hemoglobin level of <11 g/dL (reference 11–15 g/dL), and none had thrombocytopenia. All children with a positive blood smear or PCR result were treated: atovaquone/proguanil for *P. falciparum* infections, chloroquine followed by primaquine for *P. vivax* infections, and atovaquone/proguanil followed by primaquine for the mixed infection and infections with no species identified.

**Table 2:** Prevalence of infectious diseases in children adopted from Ethiopia who were seen at the University of Minnesota International Adoption Clinic, Minneapolis, Minnesota, USA, 2006–2011

Infection	No. screening results available	No. (%) positive
Intestinal parasites	217	96 (44.2)*
Tuberculosis	181	49 (27.1)†
Malaria	52	7 (13.5)
Hepatitis A virus	161	14 (8.7)
Hepatitis B virus	233	6 (2.6)
Syphilis	215	0†‡
Hepatitis C virus	219	0†‡
HIV	218	1 (0.5)‡

*Evidence of infection with >1 of the following: *Giardia intestinalis* flagellates (n = 75, 34.6%), *Blastocystis hominis* protozoa (n = 34, 15.7%), *Hymenolepsis nana* tapeworms (n = 2, 0.9%), *Dientamoeba fragilis* protozoa (n = 2, 0.9%), *Ascaris lumbricoides* roundworms (n = 2, 0.5%), or *Trichuris trichiura roundworms* (0.5%). †By tuberculin skin testing (induration >10 mm; n = 46), interferon-γ release assay (n = 1), or both (n = 2). Latent tuberculosis infection was diagnosed in 48 children. Tuberculosis disease was diagnosed initially in 1, but was later reassessed as latent tuberculosis infection; medications for disease were stopped after 4 months of treatment. ‡Initial screening tests results were positive in 2 additional children, but confirmatory tests were negative.

In addition to the malaria results, of 217 children tested for intestinal parasites, 96 (44.2%) had positive results; *Giardia intestinalis* flagellates were most common (n = 75, 34.6%), followed by *Blastocystis hominis* protozoa (n = 34, 15.7%) (Table 1). Evidence of tuberculous infection was found in 49 (27.1%) children, hepatitis A virus in 14 (8.7%), hepatitis B virus in 6 (2.6%), and HIV in 1 (0.5%) (Table 1).

**Table 1:** Value of certain characteristics or findings for predicting asymptomatic malaria parasitemia in children adopted from Ethiopia who were seen at the University of Minnesota International Adoption Clinic, Minneapolis, Minnesota, USA, 2006–2011*

Characteristic or finding	Malaria, no. (%)		Sensitivity, %	Specificity, %	PPV, %	NPV, %
	Positive, n = 7†	Negative, n = 45				
History of hepatomegaly	0	1 (2.2)	0	97.8	0	86.3
History of splenomegaly	3 (42.8)	2 (4.4)	42.8	95.6	60.0	91.5
Presence of splenomegaly during examination	2 (28.6)	3 (6.7)	28.6	92.3	40.0	89.4
Presence of hepatomegaly during examination	1 (14.3)	7 (15.6)	14.3	84.4	12.5	86.4
History of splenomegaly or presence during examination	3 (42.8)	4 (8.9)	42.8	91.1	42.9	91.1
Hemoglobin level of <11 g/dL	2 (28.6)	6 (13.3)	28.6	87.7	25.0	88.7

*NPV, negative predictive value; PPV, positive predictive value. †Children who were malaria-positive by blood smear or PCR testing.

## Conclusions

In this study, we show that 7 (13.5%) of 52 adoptees from Ethiopia who had lived in a malaria-endemic region or had hepatomegaly or splenomegaly by clinical history or on physical examination had asymptomatic malaria parasitemia. We also confirm the findings of previous studies that showed high rates of infection with intestinal parasites (particularly *G. intestinalis* flagellates) (*3*), latent tuberculosis (*3*), and hepatitis A virus (*6*) in adoptees from Ethiopia.

The rate of asymptomatic malaria parasitemia in international adoptees is not known. As adoptions increase from countries in sub-Saharan Africa and other countries with areas of potential malaria transmission, such as India and Haiti, malaria screening will need to be considered for the adopted children. On the basis of the current data, we believe reasonable first-line criteria for malaria screening in international adoptees are residence in a malaria-endemic country plus either lack of documentation that the child lived for his or her whole life in a region of that country that was malaria free (e.g., Addis Ababa in Ethiopia) or past or current splenomegaly. However, a limitation of our study is that we used essentially these criteria to screen, and the prevalence of asymptomatic malaria might have differed if we used different criteria. For example, we did not screen all children with anemia (hemoglobin level of <11 g/dL); because malaria is a leading cause of anemia in malaria-endemic areas, anemia may be a useful additional screening criterion for malaria.

For over a century, microscopy has been the standard for documentation of malaria infection in persons with clinical malaria, but PCR has greater sensitivity for detection of low-level parasitemia (*7*) and is now a field standard for detection of asymptomatic parasitemia (*8*). Multiplex PCR also enables testing and identification of all 5 *Plasmodium* species that cause disease in humans and can provide species identification at low levels of parasitemia. In our study, microscopy testing on 2 children failed to determine the malaria species, a common difficulty in persons with low-level parasitemia. Without knowing the malaria species, we had to treat the children for both *P. falciparum* and *P. vivax* infection, which involved testing for glucose-6-phosphate dehydrogenase deficiency and treatment with multiple antimalarial medications. Knowledge of the prevalent species in immigrants from a specific area can also inform public health efforts and prophylaxis planning for travelers to that area. For these reasons, PCR is likely the test of choice for detection of asymptomatic parasitemia in children adopted from malaria-endemic areas.

For 3 reasons, we treated all adopted children with asymptomatic parasitemia, whether detected by PCR or microscopy. First, a diagnosis of malaria could be missed if these children became febrile. After their adoption, many lived in areas in which malaria is rarely if ever seen, so the diagnosis of malaria might not be considered. Second, malaria can cause severe disease, so a missed diagnosis could have major health consequences for the child. Third, most antimalarial medications have a low toxicity, so treatment is not a danger to the child. 

We now use PCR to screen asymptomatic children from malaria-endemic areas. We recommend this method for centers with rapid access to PCR for all 5 human *Plasmodium* species because increased sensitivity of detection is more important than rapid detection in asymptomatically infected children. However, any symptomatic child (e.g., a child with fever) must have microscopy or rapid diagnostic testing performed immediately, because these results are typically available quickly and can guide immediate decisions regarding treatment.

In summary, this study shows that children adopted from Ethiopia who lived in malaria-endemic regions of Ethiopia or had past or current splenomegaly are at risk for asymptomatic *P. falciparum* and *P. vivax* parasitemia. The study findings support the importance of obtaining a careful history to determine malaria risk and conducting PCR screening for asymptomatic infection in children with the noted risk factors. These findings may also be relevant to children adopted from other malaria-endemic countries.
